# (*E*)-*N*-(2,3,4-Trimeth­oxy-6-methyl­benzyl­idene)aniline

**DOI:** 10.1107/S1600536808016620

**Published:** 2008-06-07

**Authors:** Hui Zhang

**Affiliations:** a Department of Chemistry and Chemical Engineering, Weifang University, Weifang 261061, People’s Republic of China

## Abstract

In the title compound, C_17_H_19_NO_3_, the C—C=N—C torsion angle between the benzene and phenyl rings is −177.3 (2)°, and the dihedral angle between the rings is 54.6 (2)°. The crystal structure is stabilized by intra­molecular hydrogen bonds and weak π–π and C—H⋯π inter­actions.

## Related literature

For related literature, see: Zhang *et al.* (2005 ▶).
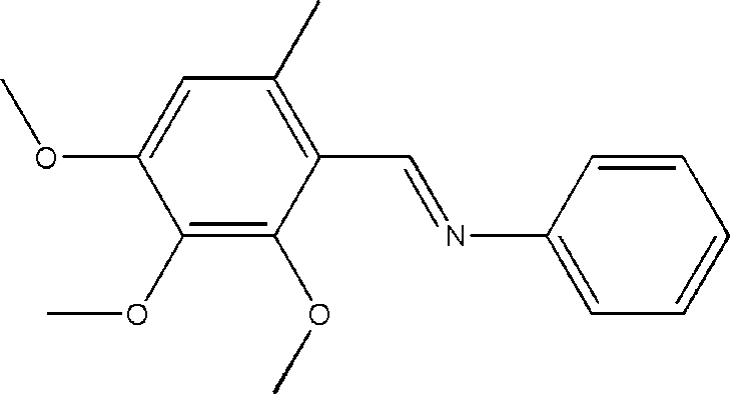


## Experimental

### 

#### Crystal data

C_17_H_19_NO_3_
*M* *_r_* = 285.33
Triclinic, 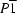

*a* = 8.3126 (13) Å
*b* = 9.9938 (17) Å
*c* = 10.8661 (19) Å
α = 110.102 (2)°
β = 111.995 (2)°
γ = 92.7000 (10)°
*V* = 769.8 (2) Å^3^
*Z* = 2
Mo *K*α radiation
μ = 0.08 mm^−1^
*T* = 298 (2) K
0.50 × 0.48 × 0.47 mm

#### Data collection

Bruker SMART CCD area-detector diffractometer
Absorption correction: multi-scan (*SADABS*; Bruker, 1997 ▶) *T*_min_ = 0.959, *T*_max_ = 0.962
3966 measured reflections
2650 independent reflections
1571 reflections with *I* > 2σ(*I*)
*R*_int_ = 0.034

#### Refinement

*R*[*F* ^2^ > 2σ(*F* ^2^)] = 0.053
*wR*(*F* ^2^) = 0.170
*S* = 1.00
2650 reflections
194 parameters
H-atom parameters constrained
Δρ_max_ = 0.19 e Å^−3^
Δρ_min_ = −0.22 e Å^−3^

### 

Data collection: *SMART* (Bruker, 1997 ▶); cell refinement: *SAINT* (Bruker, 1997 ▶); data reduction: *SAINT*; program(s) used to solve structure: *SHELXS97* (Sheldrick, 2008 ▶); program(s) used to refine structure: *SHELXL97* (Sheldrick, 2008 ▶); molecular graphics: *SHELXTL* (Sheldrick, 2008 ▶); software used to prepare material for publication: *SHELXTL*.

## Supplementary Material

Crystal structure: contains datablocks global, I. DOI: 10.1107/S1600536808016620/bx2141sup1.cif

Structure factors: contains datablocks I. DOI: 10.1107/S1600536808016620/bx2141Isup2.hkl

Additional supplementary materials:  crystallographic information; 3D view; checkCIF report

## Figures and Tables

**Table 1:** Hydrogen-bond geometry (Å, °) *Cg*2 is the centroid of the ring C12–C17.

*D*—H⋯*A*	*D*—H	H⋯*A*	*D*⋯*A*	*D*—H⋯*A*
C1—H1⋯O1	0.93	2.32	2.714 (3)	105
C8—H8*C*⋯O2	0.96	2.47	3.062 (5)	120
C9—H9*C*⋯O1	0.96	2.53	3.079 (4)	116
C10—H10*C*⋯*Cg*2^i^	0.96	2.98	3.894 (4)	160

Symmetry code: (i) 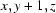.

**Table 2:** π–π interactions (Å, °) *Cg*1 is the centroid of the ring C2–C7. The offset is defined as the distance between *CgI* and the perpendicular projection of *CgJ* on ring *I*.

*CgI*⋯*CgJ*	*CgI*⋯*CgJ*	Dihedral angle	Interplanar distance	Offset
*Cg*1⋯*Cg*1^i^	4.236 (1)		3.523 (1)	2.352

Symmetry code: (i) 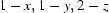.

**Fig. 1.**
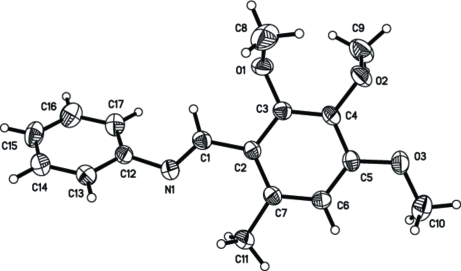
The molecular structure of (I), drawn with 30% probability ellipsoids.

## supplementary crystallographic information

### Comment

The preparation, properties and applications of Schiff bases are important in the development of coordination chemistry. In this paper, the structure of the title compound, (I), is reported. The molecular structure of (I) is illustrated in Fig. 1. The bond lengths and angles of the title compound agree with those in the related compound 2,3,4-Trimethoxy-6-methylbenzaldehyde (Zhang *et al.*, 2005), as representative example. The dihedral angle between the two phenyl rings is 125.4 (2)°. The crystal structure is stabilized by an intramolecular hydrogen bonding and weak π–π and C—H···π interactions ( Table 1 and Table 2).

### Experimental

To a solution of *p*-toluidine (0.535 g, 5 mmol) and potassium acetate (0.980 g, 10 mmol) in distilled water (10 ml), 2,3,4-Trimethoxy-6-methylbenzaldehyde (1.04 g, 5 mmol) in ethylalcohol (20 ml) was added drop by drop, the solution was stirred for 1 h at reflux temperature. The precipitate was filtered and dried. 10 mg of (I) was dissolved in 15 ml ethanol and the solution was allowed to evaporate at room temperature. Straw yellow single crystals of the title compound were formed after one week.

### Refinement

The H atoms were positioned geometrically (C—H = 0.93–0.96 Å) and refined as riding with *U*_iso_(H) = 1.2*U*_eq_(C) or 1.5 *U*_eq_(methyl C).

### Crystal data

**Table:**

C_17_H_19_NO_3_	*Z* = 2
*M**_r_* = 285.33	*F*_000_ = 304
Triclinic, *P*1	*D*_x_ = 1.231 Mg m^−^^3^
Hall symbol: -P 1	Mo *K*α radiation λ = 0.71073 Å
*a* = 8.3126 (13) Å	Cell parameters from 1209 reflections
*b* = 9.9938 (17) Å	θ = 2.4–26.5º
*c* = 10.8661 (19) Å	µ = 0.08 mm^−^^1^
α = 110.102 (2)º	*T* = 298 (2) K
β = 111.995 (2)º	Block, yellow
γ = 92.7000 (10)º	0.50 × 0.48 × 0.47 mm
*V* = 769.8 (2) Å^3^	

### Data collection

**Table:**

Bruker SMART CCD area-detector diffractometer	2650 independent reflections
Radiation source: fine-focus sealed tube	1571 reflections with *I* > 2σ(*I*)
Monochromator: graphite	*R*_int_ = 0.034
*T* = 298(2) K	θ_max_ = 25.0º
φ and ω scans	θ_min_ = 2.2º
Absorption correction: multi-scan(SADABS; Bruker, 1997)	*h* = −9→9
*T*_min_ = 0.959, *T*_max_ = 0.962	*k* = −7→11
3966 measured reflections	*l* = −12→12

### Refinement

**Table:**

Refinement on *F*^2^	Secondary atom site location: difference Fourier map
Least-squares matrix: full	Hydrogen site location: inferred from neighbouring sites
*R*[*F*^2^ > 2σ(*F*^2^)] = 0.053	H-atom parameters constrained
*wR*(*F*^2^) = 0.170	*w* = 1/[σ^2^(*F*_o_^2^) + (0.0809*P*)^2^ + 0.0591*P*] where *P* = (*F*_o_^2^ + 2*F*_c_^2^)/3
*S* = 1.00	(Δ/σ)_max_ < 0.001
2650 reflections	Δρ_max_ = 0.19 e Å^−^^3^
194 parameters	Δρ_min_ = −0.22 e Å^−^^3^
Primary atom site location: structure-invariant direct methods	Extinction correction: none

### Special details

**Table:**

Geometry. All e.s.d.'s (except the e.s.d. in the dihedral angle between two l.s. planes) are estimated using the full covariance matrix. The cell e.s.d.'s are taken into account individually in the estimation of e.s.d.'s in distances, angles and torsion angles; correlations between e.s.d.'s in cell parameters are only used when they are defined by crystal symmetry. An approximate (isotropic) treatment of cell e.s.d.'s is used for estimating e.s.d.'s involving l.s. planes.
Refinement. Refinement of *F*^2^ against ALL reflections. The weighted *R*-factor *wR* and goodness of fit *S* are based on *F*^2^, conventional *R*-factors *R* are based on *F*, with *F* set to zero for negative *F*^2^. The threshold expression of *F*^2^ > σ(*F*^2^) is used only for calculating *R*-factors(gt) *etc*. and is not relevant to the choice of reflections for refinement. *R*-factors based on *F*^2^ are statistically about twice as large as those based on *F*, and *R*- factors based on ALL data will be even larger.

### Fractional atomic coordinates and isotropic or equivalent isotropic displacement parameters (Å^2^)

**Table:**

	*x*	*y*	*z*	*U*_iso_*/*U*_eq_	
N1	0.6434 (3)	0.0882 (2)	0.7849 (2)	0.0637 (6)	
O1	0.1396 (2)	0.11706 (18)	0.64766 (19)	0.0619 (5)	
O2	0.0101 (2)	0.3729 (2)	0.67375 (19)	0.0642 (5)	
O3	0.2329 (2)	0.62688 (18)	0.78978 (19)	0.0626 (5)	
C1	0.4895 (3)	0.1089 (3)	0.7390 (3)	0.0489 (6)	
H1	0.3988	0.0268	0.6846	0.059*	
C2	0.4379 (3)	0.2505 (2)	0.7625 (2)	0.0424 (6)	
C3	0.2546 (3)	0.2493 (2)	0.7129 (2)	0.0457 (6)	
C4	0.1898 (3)	0.3749 (3)	0.7225 (2)	0.0468 (6)	
C5	0.3079 (3)	0.5078 (3)	0.7853 (2)	0.0471 (6)	
C6	0.4884 (3)	0.5112 (3)	0.8370 (2)	0.0470 (6)	
H6	0.5664	0.6004	0.8804	0.056*	
C7	0.5564 (3)	0.3853 (3)	0.8261 (2)	0.0452 (6)	
C8	0.0442 (5)	0.0901 (4)	0.7236 (4)	0.0963 (11)	
H8A	0.1254	0.0873	0.8125	0.144*	
H8B	−0.0376	−0.0015	0.6660	0.144*	
H8C	−0.0198	0.1663	0.7438	0.144*	
C9	−0.0840 (4)	0.3143 (4)	0.5226 (3)	0.0889 (11)	
H9A	−0.0245	0.3586	0.4815	0.133*	
H9B	−0.2018	0.3333	0.4977	0.133*	
H9C	−0.0900	0.2112	0.4858	0.133*	
C10	0.3479 (4)	0.7643 (3)	0.8476 (3)	0.0751 (9)	
H10A	0.4278	0.7854	0.9456	0.113*	
H10B	0.2790	0.8385	0.8448	0.113*	
H10C	0.4144	0.7616	0.7915	0.113*	
C11	0.7545 (3)	0.4004 (3)	0.8838 (3)	0.0616 (7)	
H11A	0.8096	0.5015	0.9259	0.092*	
H11B	0.7866	0.3496	0.8066	0.092*	
H11C	0.7936	0.3599	0.9558	0.092*	
C12	0.6728 (3)	−0.0566 (3)	0.7476 (3)	0.0504 (6)	
C13	0.7910 (3)	−0.0901 (3)	0.8552 (3)	0.0649 (8)	
H13	0.8417	−0.0205	0.9492	0.078*	
C14	0.8357 (4)	−0.2241 (3)	0.8266 (4)	0.0736 (8)	
H14	0.9156	−0.2451	0.9009	0.088*	
C15	0.7636 (5)	−0.3261 (3)	0.6901 (4)	0.0758 (9)	
H15	0.7945	−0.4169	0.6708	0.091*	
C16	0.6450 (4)	−0.2958 (3)	0.5803 (3)	0.0746 (9)	
H16	0.5953	−0.3660	0.4867	0.090*	
C17	0.5994 (4)	−0.1610 (3)	0.6089 (3)	0.0614 (7)	
H17	0.5190	−0.1404	0.5345	0.074*	

### Atomic displacement parameters (Å^2^)

**Table:**

	*U*^11^	*U*^22^	*U*^33^	*U*^12^	*U*^13^	*U*^23^
N1	0.0507 (14)	0.0530 (14)	0.0845 (16)	0.0168 (11)	0.0250 (12)	0.0260 (12)
O1	0.0464 (10)	0.0564 (11)	0.0774 (13)	0.0030 (8)	0.0300 (9)	0.0160 (9)
O2	0.0420 (10)	0.0785 (13)	0.0730 (13)	0.0211 (9)	0.0266 (9)	0.0264 (10)
O3	0.0661 (12)	0.0558 (11)	0.0766 (12)	0.0272 (9)	0.0343 (10)	0.0311 (9)
C1	0.0447 (15)	0.0542 (15)	0.0534 (14)	0.0107 (12)	0.0250 (12)	0.0224 (12)
C2	0.0423 (13)	0.0477 (14)	0.0424 (13)	0.0130 (11)	0.0208 (11)	0.0194 (10)
C3	0.0430 (14)	0.0489 (15)	0.0461 (13)	0.0089 (11)	0.0227 (11)	0.0150 (11)
C4	0.0398 (14)	0.0578 (16)	0.0471 (14)	0.0158 (12)	0.0220 (11)	0.0202 (11)
C5	0.0522 (15)	0.0507 (15)	0.0475 (14)	0.0196 (12)	0.0266 (12)	0.0219 (11)
C6	0.0465 (14)	0.0481 (14)	0.0461 (13)	0.0063 (11)	0.0197 (11)	0.0181 (11)
C7	0.0427 (14)	0.0535 (15)	0.0458 (13)	0.0130 (12)	0.0214 (11)	0.0230 (11)
C8	0.110 (3)	0.081 (2)	0.134 (3)	0.0123 (19)	0.084 (3)	0.046 (2)
C9	0.0557 (18)	0.108 (3)	0.075 (2)	0.0228 (18)	0.0085 (16)	0.0230 (19)
C10	0.094 (2)	0.0561 (18)	0.080 (2)	0.0248 (16)	0.0366 (18)	0.0302 (15)
C11	0.0456 (15)	0.0611 (17)	0.0775 (18)	0.0098 (12)	0.0219 (14)	0.0304 (14)
C12	0.0419 (14)	0.0499 (15)	0.0683 (17)	0.0136 (11)	0.0293 (13)	0.0256 (13)
C13	0.0525 (16)	0.0590 (17)	0.0702 (18)	0.0132 (13)	0.0153 (14)	0.0215 (14)
C14	0.0603 (18)	0.071 (2)	0.094 (2)	0.0209 (15)	0.0252 (17)	0.0437 (18)
C15	0.094 (2)	0.0582 (19)	0.104 (3)	0.0350 (17)	0.061 (2)	0.0383 (18)
C16	0.099 (2)	0.0641 (19)	0.0689 (19)	0.0214 (17)	0.0485 (18)	0.0202 (15)
C17	0.0710 (18)	0.0653 (18)	0.0662 (18)	0.0230 (14)	0.0393 (15)	0.0334 (15)

### Geometric parameters (Å, °)

**Table:**

N1—C1	1.244 (3)	C9—H9A	0.9600
N1—C12	1.422 (3)	C9—H9B	0.9600
O1—C3	1.376 (3)	C9—H9C	0.9600
O1—C8	1.416 (3)	C10—H10A	0.9600
O2—C4	1.382 (3)	C10—H10B	0.9600
O2—C9	1.409 (3)	C10—H10C	0.9600
O3—C5	1.363 (3)	C11—H11A	0.9600
O3—C10	1.423 (3)	C11—H11B	0.9600
C1—C2	1.464 (3)	C11—H11C	0.9600
C1—H1	0.9300	C12—C13	1.373 (4)
C2—C7	1.410 (3)	C12—C17	1.379 (4)
C2—C3	1.411 (3)	C13—C14	1.370 (4)
C3—C4	1.375 (3)	C13—H13	0.9300
C4—C5	1.396 (3)	C14—C15	1.355 (4)
C5—C6	1.385 (3)	C14—H14	0.9300
C6—C7	1.389 (3)	C15—C16	1.372 (4)
C6—H6	0.9300	C15—H15	0.9300
C7—C11	1.505 (3)	C16—C17	1.380 (4)
C8—H8A	0.9600	C16—H16	0.9300
C8—H8B	0.9600	C17—H17	0.9300
C8—H8C	0.9600		
			
C1—N1—C12	119.4 (2)	O2—C9—H9C	109.5
C3—O1—C8	116.2 (2)	H9A—C9—H9C	109.5
C4—O2—C9	114.82 (19)	H9B—C9—H9C	109.5
C5—O3—C10	117.8 (2)	O3—C10—H10A	109.5
N1—C1—C2	126.0 (2)	O3—C10—H10B	109.5
N1—C1—H1	117.0	H10A—C10—H10B	109.5
C2—C1—H1	117.0	O3—C10—H10C	109.5
C7—C2—C3	118.4 (2)	H10A—C10—H10C	109.5
C7—C2—C1	125.0 (2)	H10B—C10—H10C	109.5
C3—C2—C1	116.5 (2)	C7—C11—H11A	109.5
C4—C3—O1	120.0 (2)	C7—C11—H11B	109.5
C4—C3—C2	121.8 (2)	H11A—C11—H11B	109.5
O1—C3—C2	118.1 (2)	C7—C11—H11C	109.5
C3—C4—O2	121.5 (2)	H11A—C11—H11C	109.5
C3—C4—C5	119.4 (2)	H11B—C11—H11C	109.5
O2—C4—C5	119.1 (2)	C13—C12—C17	118.6 (2)
O3—C5—C6	124.8 (2)	C13—C12—N1	117.4 (2)
O3—C5—C4	115.7 (2)	C17—C12—N1	123.8 (2)
C6—C5—C4	119.5 (2)	C14—C13—C12	121.1 (3)
C5—C6—C7	122.0 (2)	C14—C13—H13	119.4
C5—C6—H6	119.0	C12—C13—H13	119.4
C7—C6—H6	119.0	C15—C14—C13	120.0 (3)
C6—C7—C2	118.9 (2)	C15—C14—H14	120.0
C6—C7—C11	117.9 (2)	C13—C14—H14	120.0
C2—C7—C11	123.2 (2)	C14—C15—C16	120.2 (3)
O1—C8—H8A	109.5	C14—C15—H15	119.9
O1—C8—H8B	109.5	C16—C15—H15	119.9
H8A—C8—H8B	109.5	C15—C16—C17	119.9 (3)
O1—C8—H8C	109.5	C15—C16—H16	120.0
H8A—C8—H8C	109.5	C17—C16—H16	120.0
H8B—C8—H8C	109.5	C12—C17—C16	120.1 (3)
O2—C9—H9A	109.5	C12—C17—H17	119.9
O2—C9—H9B	109.5	C16—C17—H17	119.9
H9A—C9—H9B	109.5		
			
C12—N1—C1—C2	−177.3 (2)	O2—C4—C5—C6	178.8 (2)
N1—C1—C2—C7	8.3 (4)	O3—C5—C6—C7	−178.4 (2)
N1—C1—C2—C3	−173.8 (2)	C4—C5—C6—C7	1.2 (3)
C8—O1—C3—C4	−70.8 (3)	C5—C6—C7—C2	−1.1 (3)
C8—O1—C3—C2	112.1 (3)	C5—C6—C7—C11	179.2 (2)
C7—C2—C3—C4	1.3 (3)	C3—C2—C7—C6	−0.2 (3)
C1—C2—C3—C4	−176.7 (2)	C1—C2—C7—C6	177.7 (2)
C7—C2—C3—O1	178.33 (19)	C3—C2—C7—C11	179.6 (2)
C1—C2—C3—O1	0.3 (3)	C1—C2—C7—C11	−2.6 (4)
O1—C3—C4—O2	3.0 (3)	C1—N1—C12—C13	−136.1 (3)
C2—C3—C4—O2	−180.0 (2)	C1—N1—C12—C17	48.6 (4)
O1—C3—C4—C5	−178.2 (2)	C17—C12—C13—C14	−0.1 (4)
C2—C3—C4—C5	−1.2 (3)	N1—C12—C13—C14	−175.7 (2)
C9—O2—C4—C3	−72.5 (3)	C12—C13—C14—C15	0.3 (4)
C9—O2—C4—C5	108.7 (3)	C13—C14—C15—C16	−0.3 (5)
C10—O3—C5—C6	2.1 (3)	C14—C15—C16—C17	0.2 (4)
C10—O3—C5—C4	−177.6 (2)	C13—C12—C17—C16	0.0 (4)
C3—C4—C5—O3	179.6 (2)	N1—C12—C17—C16	175.2 (2)
O2—C4—C5—O3	−1.6 (3)	C15—C16—C17—C12	0.0 (4)
C3—C4—C5—C6	−0.1 (3)		

### Hydrogen-bond geometry (Å, °)

**Table:**

*D*—H···*A*	*D*—H	H···*A*	*D*···*A*	*D*—H···*A*
C1—H1···O1	0.93	2.32	2.714 (3)	105
C8—H8C···O2	0.96	2.47	3.062 (5)	120
C9—H9C···O1	0.96	2.53	3.079 (4)	116
C10—H10C···Cg2^i^	0.96	2.98	3.894 (4)	160

Symmetry codes: (i) *x*, *y*+1, *z*.

### Table 2 π–π interactions (Å, °)

Cg1 is the centroid of ring C2–C7. The offset is defined as the distance between CgI and the perpendicular projection of CgJ on ring I.

**Table:**

CgI-CgJ	CgI···CgJ	Dihedral angle	Interplanar distance	Offset
Cg1-Cg1i	4.236 (1)	0	3.523 (1)	2.352

Symmetry code: (i) 1-x, 1-y, 2-z.
